# Basic Aspects and Epidemiological Studies on Leptospirosis Carried Out in Animals in Chile: A Bibliographic Review

**DOI:** 10.3390/tropicalmed8020097

**Published:** 2023-02-01

**Authors:** Lucía Azócar-Aedo

**Affiliations:** Facultad de Ciencias de la Naturaleza, Escuela de Medicina Veterinaria, Universidad San Sebastián, Sede De La Patagonia, Puerto Montt 5480000, Chile; lucia.azocara@uss.cl

**Keywords:** leptospirosis, zoonosis, epidemiology, humans, domestic animals, wild animals, Chile

## Abstract

Leptospirosis is an important zoonosis worldwide. This disease affects numerous animal species, some of them are classified as “maintenance hosts”, and others are categorized as “incidental hosts”. Humans are at risk of becoming infected by having contact with domestic and wild animals. In this paper, general aspects of the etiology and transmission of leptospirosis are addressed, data regarding the clinical presentation of the pathology in humans and animals are also presented, and the results of some epidemiological studies on leptospirosis carried out in Chile in different animal species and humans are summarized through a bibliographic review of the literature. The research on domestic canines and horses stands out in terms of their number in the country, with prevalences between 12.0% and 59.1% in dogs and from 23.3% to 65.4% in equids. Studies have been performed on domestic felines in recent years with frequencies ranging from 3.0% to 25.2%, as well as on wild animals (mainly in mammals). In pigs, cattle, sheep, and goats, the information is scarce, with little updated research dating back several decades and variable prevalence rates, which are generally high, except for in sheep. Leptospirosis is a disease of varied etiology in terms of infecting species, serovars and serogroups, which influences its epidemiology, and its prevalence is variable in different animals. An increase in the awareness given to this pathology in human and veterinary public health is required, as well as more scientific studies in Chile, to update the existing knowledge.

## 1. Introduction

Most of the human infections have an animal origin [1]. Leptospirosis is a zoonosis, which is caused by an infection from pathogenic bacteria of the genus *Leptospira* [2]. It has worldwide distribution primarily in geographical areas of tropical, subtropical, and temperate climates [3,4]. It is probably endemic in many countries with no available surveillance systems or diagnostic laboratories [5]. It is prevalent because of poor basic sanitation conditions, inadequate garbage management, poor prevention and control measures, and neglecting the disease [6].

The genus *Leptospira* belongs to the Leptospiraceae family and the order Spirochaetales [7]. They are long and thin bacteria, which are approximately 0.1 to 0.15 µm thick and 6 to 20 µm long and spiral-shaped. Like most Gram-negative bacteria, leptospires have a protein-containing outer membrane and a periplasmic flagellum which allows motility [8].

The virulence of leptospires depends on their lipopolysaccharide, which is the main recognized antigen during infection and also responsible for antigenic diversity and classification [9]. The genus *Leptospira* has been divided into three groups based on their pathogenicity: (1) saprophytic species, (2) species of intermediate pathogenicity, and (3) pathogenic species [10]. The serovar is the unit wherein the different species are cataloged, with each one having different antigenic conformations. There are more than 300 serovars, which have been classified in about 32 serogroups according to their antigenic homology [11]. Each geographical area in the world is characterized by serogroups/serovars, which were determined by the ecology of the place, and both the prevalence of the disease and the distribution of serovars vary between different countries, and even between regions within a country [12].

The precise identification and classification of the *Leptospira* genus is necessary for epidemiological and public health surveillance, since the serovars show different host specificities [13]. Each serovar is adapted to one or more mammals which act as “maintenance hosts,” harboring the bacteria without showing clinical signs, but excreting the microorganism in the urine, acting as the reservoirs [14]. After infection, leptospires appear in the blood and invade practically all tissues and organs, which would be eliminated later from the body through the action of the immune system. However, leptospires are able to colonize the renal tubules and then are excreted in the urine over a period of a few weeks to several months [15].

The “incidental hosts” are also described, wherein the infection is associated with high titers of antibodies with a short-term renal carrier state [14,16] and the development of a clinical disease at different severity levels, with clinical symptoms such as fever, signs of respiratory disease, fertility problems, and kidney and liver failure [17,18]. An animal species can act as a maintenance host for some serovars and, at the same time, can be an incidental hosts for others [19].

In a given geographical area, an animal species will be infected by serovars, which are maintained by other species [2]. In rural areas, cattle, pigs, sheep, and goats present a high risk of infection [20]. For instance, the serovar Hardjo is adapted to cattle, which are its maintenance host [2]. The Pomona serovar is associated with pigs, cattle, and wildlife, such as skunks and opossums, and the Autumnalis serovar has been associated with rodents, while the Bratislava serovar is adapted to rats, pigs, and horses [21]. In urban areas, rodents, particularly rats, are the main reservoirs of the bacteria, which harbor the serovar Icterohaemorrhagiae [22]; however, rodents can also be reservoirs for the serogroups Ballum, Autumnalis, and Copenhageni [23,24,25]. Domestic canines are maintenance and incidental hosts of the serovar Canicola [20,21].

The transmission of leptospirosis can be direct or indirect. Direct transmission happens by contact with the urine of maintenance hosts, placental transfer, or bites. The organism penetrates mucous membranes or broken skin. Indirect transmission occurs through exposure of susceptible animals or humans to environments which are contaminated with urine (soil or water) [17], considered the most common mechanism [4]. This transmission depends on several factors, such as the climatic conditions of the environment, population density, and the level of contact between maintenance and incidental hosts [23]. When excreted in the urine of infected animals, pathogenic leptospires reach the environment and survive, but do not multiply. Exposure to muddy soils or stagnant fresh water could increase the chances of infection in both humans and animals [22]. Rains, floods, high temperatures, and even the occurrence of environmental disasters have been related to leptospirosis outbreaks and are considered risk factors for disease transmission [26].

In developing countries, leptospirosis has considerable economic importance in livestock, but it is also relevant in humans, where the major burden of disease occurs in the tropical and subtropical regions [23]. Although epidemiological surveillance of leptospirosis in humans and animals is essential for its prevention worldwide, this is currently extremely limited [27].

The aims of this bibliographic review are as follows: (1) detail epidemiological and clinical aspects of leptospirosis in humans and animals; and (2) present data from some epidemiological studies or reports on leptospirosis which are carried out among humans and different animal species in Chile.

## 2. Materials and Methods

### 2.1. Study Design

This study corresponds to a bibliographical review of the literature, theoretical and narrative [28].

### 2.2. Bibliographic Search Strategy

A bibliographic search was conducted in the electronic documentary databases PubMed and ScienceDirect and via the search engine Google Scholar. There was no consideration given to the year of publication of the information [29].

Documents related to the etiology and clinical signs of leptospirosis in humans and domestic and wild animals were searched. This search was carried out both in humans and in the following animal species: domestic canines (*Canis familiaris*), domestic felines (*Felis catus*), equines (*Equus caballus*), pigs (*Sus scrofa*), bovines (*Bos taurus*), sheep (*Ovis aries*), and goats (*Capra hircus*). The keywords used were as follows: leptospirosis animals, leptospirosis humans, *Leptospira* animals, *Leptospira* humans, leptospirosis clinical signs humans, leptospirosis clinical signs animals, *Leptospira* AND animals, *Leptospira* AND humans, *Leptospira* OR leptospirosis AND animals, *Leptospira* OR leptospirosis AND humans, as well as “leptospirosis dogs”, “leptospirosis cats”, “leptospirosis horses”, “leptospirosis cattle”, “leptospirosis sheep”, “leptospirosis goats”, “leptospirosis pigs”, “leptospirosis wild animals”.

Data on the epidemiology of the disease in Chile in humans and animals were also collected, specifically prevalence obtained with indirect diagnostic tests (serology, in particular, the microscopic agglutination test (MAT) or enzyme-linked immunosorbent assay (ELISA) and direct diagnostic tests (such as polymerase chain reaction and bacteriological culture). The keywords used were leptospirosis animal prevalence Chile, leptospirosis human prevalence Chile, *Leptospira* animal prevalence Chile, *Leptospira* human prevalence Chile, *Leptospira* AND animals AND prevalence Chile, *Leptospira* AND humans AND prevalence Chile.

### 2.3. Inclusion and Exclusion Criteria

Consideration was given to bibliographic reviews related to the etiology, clinical signs, and epidemiology of leptospirosis in humans and animals. Epidemiological studies of cross-sectional design or cases and controls were included, which must specify the frequency of presentation or prevalence or incidence of leptospirosis in Chile in humans, domestic and wild animals, as well as reports of the disease from government organizations related to human and animal health.

The type of documents considered were research articles, abstracts of articles, government official reports, and books.

Conference presentations and information from web pages from unspecified sources and year of publication were excluded.

### 2.4. Data Extraction

Information regarding the etiology and clinical signs of the disease in people as well as in domestic and wild animals was collected and summarized.

For epidemiologic data, in all the included studies, the animal species in which the study was carried out, and the geographical area (country, city, and geographical location), the diagnostic test used, reported prevalence and/or incidence rate, and frequently detected serovars were recorded. In articles that used MAT as a diagnostic test, the serovar/serogroup that caused the serological reaction, as well as the antibody titer, was compiled.

### 2.5. Data Analysis

The data were presented in a qualitative, narrative, and descriptive way [30,31].

## 3. Results

### 3.1. Bibliographic Review

#### 3.1.1. Clinical Aspects and Epidemiological Data on Human Leptospirosis in Chile

Human leptospirosis is always incidental [27]. The disease varies from a subclinical infection to even a severe multi-organ syndrome with high mortality [32]. An anicteric form similar to influenza may occur, with symptoms including fever, myalgia, headache, abdominal pain, nonproductive cough, and conjunctival suffusion. In 5–10% of the cases, jaundice or hepatonephrotic syndrome is present, which is also known as “Weil disease,” and is characterized by severe multi-organ dysfunction [9], wherein myocarditis, hemorrhages, uveitis, and multi-organ failure have also been described, possibly leading to death [33]. Leptospirosis is often misdiagnosed as aseptic meningitis, influenza, liver disease, fever of unknown origin, or tropical diseases, such as malaria or yellow fever, and other pathologies as infection by hantavirus, rickettsiosis, borreliosis, brucellosis, or toxoplasmosis because of the variety of the symptoms seen in people [2,32].

The transmission of leptospirosis from animals to humans is more frequent in occupationally exposed groups, such as agricultural workers, people who work with livestock, veterinarians, tourists, and pet owners [33]. Cases are detected worldwide, but are more frequent in rural and urban environments, which highlights the prevalence reported in Latin America and mainly in South American countries [9]. Although exact epidemiological data are scarce, most of the reported cases have severe clinical manifestations with mortality, which is greater than 10%, and worldwide, around 500.000 cases per year are estimated [2].

In Chile, leptospirosis is included among the human diseases which must be reported to the Ministry of Health [34]. During the years 2003 to 2009, cases were reported in the Maule Region, Bío-Bío Region, Los Lagos Region, Valparaíso Region, and Metropolitan Region (Table 1). The serovar was determined in 91.4% of the positive samples, with the most predominant being Icterohaemorrhagiae (42%), followed by Georgia (17.4%), and Canicola [35]. In the period between the years 2013 and 2017, the areas with high incidence rates were Maule region, Ñuble region, and the Bío Bío region (Table 1). The most frequent serovars were Icterohaemorrhagiae, Australis, Hardjo, Grippotyphosa, Canícola, and Georgia [36].

In 2021, the Ñuble region had the highest incidence rate with the presentation of two cases, which corresponded to a rate of 0.39 per 100.000 inhabitants. The regions of Valparaíso and Biobío also presented two cases, with an incidence rate of 0.10 and 0.12 per 100.000 people, respectively (Table 1). In the period 2018 to 2021, the prevalent serovars were mainly Australis, Cynopteri, and Grippotyphosa. In the last two years, the Australis and Grippotyphosa serovars predominated [37].

#### 3.1.2. Epidemiological and Clinical Aspects of Leptospirosis in Animals

Leptospires have been isolated from more than 60 species of mammals, including reptiles, amphibians, fish, and invertebrates [38]. Species considered as important sources of infection for humans are small mammals, particularly wild and peridomestic rodents (rats and mice), insectivorous mammals (shrews and hedgehogs), and domestic animals (cattle, pigs, sheep, goats, horses, and canines) [15].

Leptospirosis is a systemic disease in dogs, cattle, pigs, and horses [7]. An acute form of the disease may occur, which is characterized by an icterohemorrhagic syndrome, but with variable clinical signs. In cattle, sheep, goats, pigs, and horses, the infection causes abortions and infertility or reproductive problems, generating significant animal and economic losses [17].

Vaccines for veterinary use are suspensions of one or more inactivated pathogenic strains of *Leptospira* which are available worldwide for cattle, pigs, and dogs [39]. With regard to the efficacy of vaccines, it has been observed, for example, that the ideal scenario is to vaccinate cattle before possible exposure and continue the immunization on an annual basis. For a vaccination program to work, it is necessary to carry out epidemiological studies wherein the incidence of the different serovars/serogroups of *Leptospira* in a given population would be evaluated [7].

##### Domestic Dogs

These animals are maintenance and incidental hosts of *Leptospira* in urban and rural environments. The infection caused by the serovar Canicola is the most common. Contact with the urine of carrier dogs is the main route of transmission. Owing to the behavioral habits of dogs, such as sniffing and licking other canines, interspecies transmission is enhanced, with stray dogs being an important source of infection [40]. The infection begins with clinical signs, such as vomiting, depression, anorexia, weakness, and fever. With the serovar Canicola, a subacute and acute disease develops. The subacute form commonly manifests itself with fever, depression, anorexia, and nephritis, and in the acute form (known as “Stuttgart Disease”) vomiting is also observed, which can rapidly progress to dehydration and even cause death [21].

Canine vaccines confer protection against Canicola and Icterohaemorrhagiae serovars and sometimes include other specific serovars, which depends on the geographical area [41]. It is recommended that dogs be vaccinated annually, although post-vaccination antibody titers are usually low (1:100 to 1:400). Vaccinated animals will have serological reactions to diagnostic tests which detect anti-*Leptospira* antibodies [17,42].

##### Domestic Cats

Domestic cats are a possible risk factor for disease transmission [43]. There is relatively little information on feline leptospirosis, specifically on the specific characteristics of the disease, the clinical utility of diagnostic tests, and treatment options [44], although some authors indicate that the condition is not very different from dogs [4], which have mild clinical signs despite the presence of leptospiremia and leprospiruria. The clinical signs include depression, anorexia, weight loss, polyuria, polydipsia, ascites, vomiting, diarrhea, body pain, and kidney and liver disease. Stray cats are at increased risk of infection since they are in close contact with potential *Leptospira* maintenance hosts. Additionally, felines living in rural areas can also become infected by having contact with livestock [43]. Some epidemiological studies using PCR as a diagnostic test have established the renal carrier status of *Leptospira* species, which confirms that felines could be reservoirs of the bacteria and a possible risk factor for human infection [43,45].

##### Cattle

In cattle, the main serovars of *Leptospira,* which are described in infections worldwide, are Hardjo, Pomona, Canicola, Icterhaemorrhagiae, and Grippotyphosa. Cattle are the only known reservoir of the Hardjo serovar, which causes reproductive problems. There is generally no previous clinical evidence of disease in the herd until the onset of these conditions [46]. A high prevalence of infection was found (75.0% at the herd level and 44.2% at the animal level), with a predominance of seropositivity for the Sejroe serogroup (80.3%) in a systematic review on bovine leptospirosis in Latin America [47]. Worldwide, leptospirosis has been reported as one of the main causes of reproductive disorders in cattle by causing abortions [48]. Abortions may be the only clinical sign of leptospirosis in a herd depending on the stage of pregnancy, which usually occurs in the last third of gestation. There may also be congenital infections in which animals are born dead or weak and with degenerative pathologies in the liver, kidney, or both. If these animals survive, they can become chronic carriers of the bacteria [4].

It is also described that clinical leptospirosis in cattle varies from inapparent infections to acute cases, which present non-pathognomonic signs since the severity of the disease depends on age, immunity, and the infecting dose of the bacteria. The most reported clinical signs are depression, anorexia, conjunctival suffusion, diarrhea, and fever. In lactating animals, agalactia may occur after 2 to 3 weeks [4]. Disease control is carried out according to the identification and treatment of apparently healthy urinary carriers, quarantine for recently acquired animals, antibiotic treatment of those infected, and routine immunization with commercial vaccines which contain the serovars circulating in the geographic location where the animals are found [48].

##### Small Ruminants (Sheep and Goats)

Knowledge about leptospirosis in small ruminants (sheep and goats) is still scarce, but some studies provide evidence that the infection is frequent and a great diversity of circulating serovars is described, with a predominance of Hardjo [49]. The other serovars/serogroups implicated as incidental are Pomona, Ballum, Icterohaemorrhagiae, and Grippotyphosa [50]. Small ruminants may play a role in the epidemiology of the disease by a possible shedding of the bacteria through urine, although most infections are asymptomatic [51].

Leptospirosis should be considered a probable cause of abortion in sheep and goats according to the information which was provided in studies carried out in Brazil, Argentina, Bolivia, Guyana, Peru, Ecuador, Colombia, Venezuela, and Chile [52]. Subclinical infection is mainly characterized by reproductive disorders, such as infertility, increased number of services per conception, and prolonged intervals between parturitions, abortions, stillbirths, and the birth of weak lambs/kids [53]. Acute infection produces clinical signs such as depression, anorexia, fever, and hemoglobinemia and hemoglobinuria [4].

##### Pigs

In pigs, the Bratislava serovar has been associated with reproductive problems, such as abortion, infertility, and birth of weak piglets. These animals may be maintenance hosts for serovars Pomona, Muenchen, Tarassovi, and Mitis, and they may also be incidental hosts for Icterohaemorrhagiae, Canicola, and Hardjo [54]. In acute leptospirosis, clinical signs, such as anorexia, conjunctival suffusion, jaundice, and fever, are described. Additionally, there may be cases of abortions or neonatal illness. If the infection occurs in the first third of gestation, the fetuses usually recover; however, if it occurs in the last third of gestation, abortions occur, with the leptospires being found in the fetus, placenta, and fetal membranes [4]. However, the disease begins usually with fever and the occurrence of reproductive problems [55].

##### Horses

The clinical features of equine leptospirosis are similar to those which are in seen in other animals, with depression, anorexia, and fever in the mild form of the disease. In the most severe forms, a wide variety of signs are described, which includes conjunctival suffusion, jaundice, anemia, and petechial hemorrhages in mucous membranes [4]. As with the other animals, an infection in pregnant mares can result in placentitis, abortions, or stillbirths [56]. Abortions occur in advanced gestations, typically without previous clinical signs. In a small number of cases, premature or weak foals are born [57]. However, not all infected animals develop acute disease, and subclinical infections are very common in endemic regions [58]. It is also described that two to eight months after the initial infection, a large proportion of animals (>45% in some reports) develop periodic ophthalmia, with iridocyclitis and uveitis, a condition also known as “head blindness moon” [4].

Almost all epidemiological studies on equine leptospirosis are based on serology, and the frequencies of presentation of the disease vary depending on the geographical region. There is also variability in the serovars/serogroups that are involved in the infection. However, one of the most frequently reported serovars is Icterohaemorrhagiae, which usually leads to acute systemic illness [57].

##### Wild Animals

Leptospirosis has been described in almost all warm-blooded animals worldwide. A lot of the information which exists in wild animals has been collected from captive species [4]. The role of wild animals as a source of infection in cattle and humans is unknown, but it was taken into consideration that these animals can act as hosts for serovars/serogroups of leptospires which can infect domestic animals [59].

According to Faine [4], it is unknown if birds in natural conditions can acquire *Leptospira* infection; however, they produce antibodies against the bacteria. Embryonated eggs from domestic chickens can be infected by chorioallantoic inoculation from days 9–12, and petechial hemorrhages can be seen within 48–72 h [60,61].

A systematic review of published studies on leptospirosis in Latin America found that in the Mammalia class, the predominant *Leptospira* serogroups were Icterohaemorrhagiae and Australis, and for the orders Carnivora and Rodentia, only Icterohaemorrhagiae was observed. However, the study described that leptospirosis was widespread in wildlife in all biomes of Latin America [59]. In another systematic review carried out by Browne [62], 86 studies registered over 80 species affected by leptospirosis in the Americas, mostly in the USA and Brazil, and most of the wildlife studied was terrestrial, particularly boars and racoons, with some reports on aquatic animals such as sea lions, manatees and reptiles (boas and crocodiles); the most common serovars reported were Icterohaemorrhagiae, Pomona, Grippotyphosa, and Canicola. More research is needed to determine the role of these animals in the epidemiology of leptospirosis and its impact on public health [59].

## 4. Data from Epidemiological Studies on Leptospirosis Carried out in Chile in Different Animal Species

In Table 2, data are described about epidemiological studies on leptospirosis in different animal species in Chile, specifically domestic dogs, domestic cats, horses, cattle, sheep, goats, pigs, and wild animals. The author(s) of the studies and the year of publication, as well as the animal species, the geographic location, the diagnostic test used, the sample size, the number of positive animals reported, the prevalence, some serovars reported, and cut-off values for antibody titers are provided.

## 5. Discussion

This study is a bibliographic review of the scientific literature that describes basic aspects of the etiology of leptospirosis and provides a brief revision of the clinical signs of the disease in humans and animals, as well as epidemiological aspects focused on the main results of reports on leptospirosis in people and studies conducted on different animal species in Chile, specifically domestic dogs and cats, cattle, sheep, goats, pigs, horses, and wild animals, with the results of prevalence studies and most frequent serovars/serogroups. This review was carried out in Chile due to the lack of knowledge about epidemiology and clinical aspects of the disease in this country, where leptospirosis is an important pathology for human and animal health.

In *Leptospira* infection, it is remarkable that intraspecies and interspecies transmission is dependent on the reservoir host animals, in which the bacteria replicate and are shed in urine over time, the persistence of spirochetes in the environment, and the subsequent human–animal–environmental interactions [90]. Therefore, leptospirosis is a zoonosis that presents a complex epidemiology in which a variety of domestic and wild animal species are involved; hence, human and veterinary medicine must increase awareness and implement prevention measures [44]. It is important to perform studies, either bibliographic reviews, systematic reviews, meta-analyses, or epidemiological surveys with different designs, such as cross-sectional, case–control, and cohort studies, to maintain a constant update on the existing knowledge regarding the disease. As a result of the intensification of the interaction between animals and humans in natural environments, leptospirosis is considered an emerging zoonosis of global public interest [33]. For this reason, it is an excellent example of “One Health” [91]. This approach is essential, since in leptospirosis human infection invariably results from exposure to animals or environments, which are contaminated by infected animals [33].

As mentioned above, human leptospirosis in Chile is a disease of mandatory declaration to the Ministry of Health. The notification of leptospirosis is immediate, that is, in the event of a suspected case of the disease, it must be notified in the place where it was detected, and the clinical physician must inform the government authorities [34]. However, in the country, the epidemiological information referring to leptospirosis in people is restricted to the biannual or annual reports by the Ministry of Health, and case notifications are generally not numerous. However, there are cases of the disease in different regions of the country (Table 1). Diagnostic difficulties at the medical and laboratory level contribute to the under-diagnosis of leptospirosis in many countries [23]. When there is a possible case, early clinical suspicion allows a better prognosis. For the public health specialists, knowledge of the epidemiology of the disease can help guide health decision-making at the local or regional level. Additional measures would be creating public health policies, spatial planning policies, wastewater management, and control of wild or stray animals, which are factors influencing the emergence of the disease [22]. Is necessary to perform a study in Chile at the national level to determine the seropositivity of *Leptospira* of the population in general and to focus on risk groups, such as people with occupational or recreational exposure to the bacteria.

It was found that in Chile, there are epidemiological studies on leptospirosis conducted in different animal species, such as domestic canines and felines, ruminants (cattle, sheep, and goats), horses, pigs, and wild animals. These investigations mostly used serology and MAT as a diagnostic test; therefore, the reported prevalence rates correspond to the “seropositivity” or “seroprevalence,” with variable frequencies according to the speciesThe surveys are conducted in different geographical areas, cities, or even regions throughout the country. Regarding the number of studies, those carried out on domestic canines and equines stand out because of their number and updates. In dogs, eight studies were retrieved [63,64,65,66,67,68,69,70], which were published between the years 1975 and 2022, mostly in southern Chile, in the Araucanía, Los Ríos, and Los Lagos regions. The seroprevalences varied from 59.1% [63] to 12.0% [70]. Only one study used immunofluorescence, resulting in a prevalence of 5.5% [65].

Five investigations on horses are from dates from 1975 to 2021 [63,78,79,80,81], with variable results and seroprevalences in a range of 23.1% [79] to 65.4% [80]. Moreover, it is remarkable that equines with different functions have been studied, including army horses and working horses [79], polo players [78], and draft horses from indigenous communities [81]. All documents found used MAT in the diagnosis of *Leptospira* seropositivity. These studies were carried out in southern Chile, in the Araucanía Region and Bío Bío region, as well as in Central Chile. The prevalence rates reported in general are high, preventive measures should be taken, and the association between serological reactivity and the possible presence of clinical signs must be determined, given that leptospirosis in horses is known to induce ocular disease, such as periodic ophthalmia and reproductive problems [56,57,58].

In domestic cats, only three studies were found, which were all conducted in southern Chile (Los Ríos and Los Lagos regions). Two are articles published in scientific journals [45,72]. These investigations obtained varied results and are different in terms of the diagnostic test used. In the study by Azócar-Aedo [72], using MAT, a general prevalence of 8.1% was determined; however, in feline samples in urban areas, the seroprevalence was 3.1%, whereas in those from rural areas the seroprevalence was 25.0%, which reflects that in *Leptospira* infection, the environment where the animals live can influence the frequency of presentation. Noteworthily, in the investigation by Dorsch et al. [45], molecular techniques (polymerase chain reaction) were used, highlighting the finding of positive urine samples, which could indicate a possible renal elimination of the bacteria by cats. Conversely, in the study by Toro [71] ELISA was used as a diagnostic test, determining a prevalence of 20.0%. These findings emphasize the need for more research on leptospirosis in domestic cats due to the potential for the zoonotic transmission of the infection, which has already been proven in some studies [92,93]. In a recently published meta-analysis that included 93 epidemiologic studies, a global prevalence of leptospirosis in domestic cats of 11.09% was established in studies using indirect diagnostic tests, and a prevalence of 9.22% was reported in publications that used direct diagnostic tests for *Leptospira* [29].

Information on leptospirosis in ruminants in Chile is limited, with three studies in cattle [63,73,74], two in sheep [63,75], and one in goats [63]. MAT was used in all the studies. High prevalence rates in cattle were determined, with seroprevalences of 44.9% and higher rates in individuals and herds, that is, 75.0%, which is a remarkable result considering the consequences in terms of reproductive system disorders, loss of offspring due to abortions, and thus economic losses in affected herds [74]. Preventive measures such as vaccination against *Leptospira* must be considered. In the country, leptospirosis is not included within cattle diseases under official government control, but depending on future research results, *Leptospira* infection could also be considered. Research interest in leptospirosis in ruminants must be increased. The seroprevalences found in sheep were low, 7.4% [63] and 5.7% [75], but in goats higher seroprevalences were detected (24.8%) [63].

In pigs, in general, few studies were found in Chile, dating back to several years ago. The research studies carried out by Zamora et al. [63], Zamora et al. [76], and Riedemann and Zamora [82] stand out. These studies were conducted in southern Chile (Los Ríos Region) and seroprevalences ranged from 16.0% [82] to 69.9% [63] using MAT. In Chile, pig production is carried out on a family basis, with limited access to productive resources, for the purpose of promoting the basic family economy for food security [94]. Additionally, there is an industrial production with farms that include a large number of animals, and these are destined for slaughter for domestic consumption or for exportation [95]. It would be interesting to achieve epidemiological studies in both forms of breeding to update knowledge regarding the infection and be aware of possible cases that could have an adverse effect on the swine industry.

The research on leptospirosis in wildlife in Chile stands out due to the number of publications, the various geographical areas where the studies have been conducted, and the different species under study. Some investigations date back to several decades ago, in which the main focus was wild rodents [82,83]; with prevalence rates of 22.04%–36.01% using MAT and immunohistochemical staining as diagnostic tests. More up-to-date studies have covered other species, such as *Neovison vison* [84], *Octodon degus* and Darwin’s Pericote [85], *Spheniscus magellanicus* [86], Culpeo foxes, Chilla fox [87], *Licalopex culpaeus* [88], and *Neovison vison* [89]. In these papers, the frequencies of presentation of leptospirosis have been variable, which depends on the form of presentation of the bacterium in each animal species. It would be important to continue investigating the presence of seropositivity or infection in other wild animals, either living in the wild or being cared for in wildlife rehabilitation centers that concentrate on different species all in the same place, to generalize an epidemiological description according to affected individuals, with the possibility of exploring clinical signs consistent with the disease.

In Chile, epidemiological studies on leptospirosis are conducted on various animals, with some species receiving more attention than others, such as domestic canines, equines, and wildlife. However, few studies have been found on species such as domestic cats, ruminants, and pigs, which is why more research is needed. Conversely, most of the studies have been conducted in southern Chile, specifically in the regions of Araucanía, Los Ríos, and Los Lagos, for which it is necessary to broaden the geographical area under study. Considering, for example, that Chile presents different types of climatic conditions [96] and given the survival capacity of leptospires in areas with temperate climates and high humidity [23], investigations should be conducted in the north and central areas of the country.

## 6. Conclusions

Leptospirosis is a disease with varied etiology in terms of infecting species, serovars, and serogroups, which influence its epidemiology. Its prevalence is also variable in different animals. In Chile, cases occur in both humans and in domestic and wild animal species. Almost all research in animals corresponds to cross-sectional studies in southern Chile by using the MAT, PCR, and sometimes immunofluorescence and culture as diagnostic tests. In humans, the data are extracted from reports of the disease at the country level.

Taking into consideration its relevance in epidemiology and public health, an increase in the awareness, which was given to leptospirosis in human and veterinary public health, is needed and more scientific studies in Chile are required to update the existing knowledge. The design and execution of observational case–control and cohort studies in both people and animals are recommended, considering the link between animal and human infection and the zoonotic potential of the disease, including the “One Health” approach, which applies for this pathology.

## Figures and Tables

**Table 1:** Epidemiological data on leptospirosis in humans in Chile.

Authors/Year	Period (Years)	Geographic Location	Reported Incidence
Martínez et al. (2012) [36].	Years 2003 to 2009	Chile	0.13 cases per 100.000 people
	Years 2003 to 2009	Maule region	3.3 cases per 100.000 inhabitants
	Years 2003 to 2009	Bío Bío region	2.3 cases per 100.000 people
	Years 2003 to 2009	Los Lagos region	1.8 cases per 100.000 inhabitants
	Years 2003 to 2009	Valparaíso region	1.4 cases per 100.000 people
	Years 2003 to 2009	Metropolitan region	0.3 cases per 100.000 inhabitants
MINSAL (2018) [35].	Years 2013 to 2017	Chile	0.1 cases per 100.000 people
	Years 2013 to 2017	Valparaiso region	0.1 cases per 100.000 people
	Years 2013 to 2017	Maule region	0.4 cases per 100.000 inhabitants
	Years 2013 to 2017	Ñuble region	0,6 cases per 100.000 people
	Years 2013 to 2017	Bío Bío region	0.4 cases per 100.000 inhabitants
	Years 2013 to 2017	La Araucanía region	0.1 cases per 100.000 people
	Years 2013 to 2017	Los Ríos region	0.2 cases per 100.000 inhabitants
	Years 2013 to 2017	Los Lagos region	0.1 cases per 100.000 people
MINSAL (2021) [37].	Years 2012 to 2021	Ñuble region	0.39 per 100.000 inhabitants
	Years 2012 to 2021	Valparaíso region	0.10 per 10.000 people
	Years 2012 to 2021	Bío Bío region	0.12 per 100.000 inhabitants

**Table 2:** Some epidemiological studies on leptospirosis carried out in animals in Chile.

Authors/Year	Animal Species	Geographic Location	Diagnostic Test	Sample Size	Positive Animals	Reported Prevalence	Some Serovars Reported	Cut off Antibody Titers
Zamora et al. (1975) [63]	Domestic dog	Southern Chile	MAT	N/I^1^	N/I^1^	59.1%	Hebdomadis, Pomona.	N/I^1^
Pineda et al. (1993) [64]	Domestic dogs	Chillán (south central Chile)	MAT	60	23	38.3%	Canicola, Sejroe.	1:100
Silva and Riedemann (2007) [65]	Domestic dogs	Valdivia (southern Chile)	MAT	400	59	14.8% (MAT)	Canicola, Icterohaemorrhagiae, Ballum, Hardjo, Autumnalis, Pomona.	1:100
			Indirect Immunofluorescence (IFI)	50	5	5.0% (IFI)	N/A^2^	N/A^2^
Tuemmers et al. (2013) [66]	Domestic dogs	Temuco (southern Chile)	ELISA	400	85	21.3%	N/A^2^	N/A^2^
Lelu et al. (2015) [67]	Domestic dogs	Los Ríos region (southern Chile)	MAT	247	62	25.1%	Australis, Bratislava, Icterohaemorrhagiae, Markanso, Alexi, Pyrogenes, Wolfii.	1:100
Mercado (2017) [68]	Domestic dogs	La Pintana (central Chile)	MAT	119	15	12.6%	Canicola, Ballum, Tarassovi.	1:100
Azócar-Aedo et al. (2018) [69]	Domestic dogs	Los Ríos region (southern Chile)	MAT	50	6	12.0% (serological conversion rate)	Coagglutinations between different serovars.	1:100
Azócar-Aedo and Monti (2022) [70]	Domestic dogs	Los Ríos and Los Lagos regions (southern Chile)	MAT	406 urban animals	50 urban animals	12.3% urban areas.	Canicola, Pomona, Autumnalis, Pyrogenes, Icterohaemorrhagiae, Ballum, Grypotyphosa (urban areas)	1:100
				300 rural animals	36 rural animals	12.0% rural areas.	Canicola, Pomona, Autumnalis, Hardjo, Ballum (rural areas)	
Toro (2002) [71]	Domestic cats	Concepción (south central Chile)	ELISA	20	4	20.0%	N/A^2^	N/A^2^
Azócar-Aedo et al. (2014) [72]	Domestic cats	Los Ríos and Los Lagos regions (southern Chile)	MAT	96 urban animals	3 urban animals	1.8% urban areas	Canicola, Autumnalis (urban areas).	1:100
				28 rural animals	7 rural animals	25.2% rural areas	Canicola, Autumnalis, Grippothyphosa, Bataviae (rural areas).	
Dorch et al. (2020) [45]	Domestic cats	Valdivia (southern Chile)	PCR urine, culture, urine	231	36	15.6% (leptospiruria).	N/A^2^	N/A^2^
				231	7	3.0% (cats with positive culture).		
Zamora et al. (1975) [63]	Cattle	Southern Chile	MAT	N/I^1^	N/I^1^	59.1%	Hebdomadis, Pomona.	N/I^1^
Zamora et al. (1991) [73]	Cattle	Valdivia (slaughter plant) (southern Chile)	MAT	N/I^1^	N/I^1^	44.9%	Hardjo, Pomona, Tarassovi.	1:100
Salgado et al. (2014) [74]	Cattle	Smallholder dairy farms in Los Ríos region (southern Chile)	MAT	79 herds	52 herds	75.0% (herd prevalence)	Hardjo.	1:100
Zamora et al. (1975) [63]	Sheep	Southern Chile	MAT	N/I^1^	N/I^1^	7.4%	Copenhageni.	N/I^1^
Zamora et al. (1999) [75]	Sheep	Los Lagos region (southern Chile)	MAT	629	36	5.7%	Icterohaemorrhagiae, Autumnalis, Hardjo.	1:100
Zamora et al. (1975) [63]	Goats	Southern Chile	MAT	N/I^1^	N/I^1^	24.8%	Canicola.	N/I^1^
Zamora et al. (1968) [76]	Pigs	Valdivia (southern Chile)	MAT	N/I^1^	N/I^1^	37.8%	Hardjo, Pomona.	1:100
Zamora et al. (1975) [63]	Pigs	Southern Chile	MAT	N/I^1^	N/I^1^	69.9%	Pomona, Icterohaemorrhagiae, Canicola, Sentot.	N/I^1^
Riedemann y Zamora (1990) [77]	Pigs	Valdivia (southern Chile)	MAT	100	16	16.0%	Icterohaemorrhagiae, Hardjo.	N/I^1^
Zamora et al. (1975) [63]	Horses	Southern Chile	MAT	N/I^1^	N/I^1^	48.5%	Copenhageni, Canicola, Poi.	N/I^1^
Bay-Schmith (2004) [78]	Horses	Bío-Bío region (southern Chile)	MAT	108	52	48.1%	Icterohaemorrhagiae, Canicola, Pomona, Hardjo, Ballum.	N/I^1^
Tadich et al. (2015) [79]	Horses	Central Chile	MAT	160 working horses	49 working horses	30.6% working horses	Ballum, Canicola (working horses).	1:100
				266 army horses	62 army horses	23.3% army horses	Autumnalis, Ballum (army horses).	
Troncoso et al. (2013) [80]	Horses	Linares (Central Chile)	MAT	55	36	65.4%	Autumnalis, Bratislava, Canicola, Copenhageni, Hardjo.	1:100
Tuemmers et al. (2021) [81]	Horses	La Araucanía Region (southern Chile)	MAT	100	35	35.0%	Canicola, Grippothyphosa, Hardjo, Pomona.	N/I^1^
Riedemann et al. (1994) [82]	*A.olivaceus, A.longipilis, R.rattus and R.norvergicus*	Rural area of Valdivia (southern Chile)	MAT	116	26	22.0%	Hardjo, Pomona.	1:25
Zamora and Riedemann (1995) [83]	*Wild rodents*	Rural area of Valdivia (southern Chile)	Inmunochemical staining	368	133	36.1%	N/A^2^	N/A^2^
Barros et al. (2014) [84]	*Neovison vison*	Southern Chilean districts	PCR	57	31	55.6%	N/A^2^	N/A^2^
		(Los Ríos, Los Lagos, and Aysén regions)						
Correa et al. (2017) [85]	*Octodon degus,* Darwin’s Pericote (*Phyllotis darwini)*	Metropolitan region (Amancay, Rinconada, Lonquén, Santiago) (central Chile)	PCR	N/I^1^	N/I^1^	33.0%	N/A^2^	N/A^2^
Acosta et al. (2019) [86]	*Spheniscus magellanicus*	Magdalena Island (southern Chile)	MAT	132	0	0%	N/A^2^	1:100
Moya et al. (2019) [87]	Culpeo foxes (*Pseudalopex culpaeus lycoides)*	Tierra del Fuego (southern Chile)	MAT	15	3	20.0%	Ballum, Australis, Antumnalis, Borricana, Icterohaemorragiae, Autumnalis	1:50
	Chilla fox (*Pseudalopex griseus*)			12	1	8.3%		
Galarce et al. (2021) [88]	*Licalopex culpaeus*	Two zoos and four wildlife rehabilitation centers (central Chile)	MAT	13	1	7.6%	Javanica.	1:50
Salgado et al. (2021) [89]	*Neovison vison*	Los Ríos Region (southern Chile)	PCR	45	4	8.8%	N/A^2^	N/A^2^

N/I^1^: No Information. N/A^2^: Not Applicable.

## Data Availability

Data is contained within the article because the study design is a bibliographic review.
